# Patients with isolated posterior cruciate ligament rupture had a higher posterior intercondylar eminence

**DOI:** 10.1186/s12891-022-05189-w

**Published:** 2022-03-23

**Authors:** Shi Weili, Meng Qingyang, Chen Nayun, Ma Yong, Yang Yuping, Liu Ping, Ao Yingfang, Gong Xi

**Affiliations:** Department of Sports Medicine, Peking University Third Hospital. Institute of Sports Medicine of Peking University. Beijing Key Laboratory of Sports Injuries, Beijing, China

**Keywords:** Posterior cruciate ligament, MRI, Posterior intercondylar eminence, PCL risk factors

## Abstract

**Background:**

To evaluate the anatomic geometry of the posterior intercondylar eminence and its association with PCL injury risk.

**Methods:**

Patients who underwent primary PCL reconstruction from 2015 to 2018 were retrospectively analyzed. The control group included inpatients diagnosed with ACL rupture because of a sports-related accident during the same period, matched by age, gender, height, weight, and side of injury. Measurements of the height of the apex of the posterior intercondylar eminence (HPIE), the slope length (SLPIE) and the slope angle (SAPIE) of the posterior intercondylar eminence were performed using conventional MRI scans assessed by 2 blinded, independent raters. Intraclass correlation coefficients (ICCs) was used to evaluate the consistency of measurement results. Independent sample t tests, Chi-square tests, and logistic analyses were used to compare the two group, with *P* < 0.05 considered statistically significant.

**Results:**

Fifty-five patients with PCL rupture met the inclusion criteria and 55 PCL-intact matched controls were included. There were no significant differences between the groups in gender (*P* = 1.000), limb side (*P* = 0.848), age (*P* = 0.291), BMI (*P* = 0.444) or height (*P* = 0.290). Inter-observer reproducibility was excellent agreement in HPIE, SLPIE and SAPIE of case and control groups (ICC: HPIE = 0.81, SLPIE = 0.77, SAPIE = 0.85). Patients with PCL rupture had significantly greater HPIE, SAPIE (both *P* < 0.001), and SLPIE (*P* < 0.05) than PCL-intact patients. The multivariable analysis showed that HPIE (OR, 1.62 [95% CI, 1.24–2.11], *P* < 0.001) and SAPIE (OR, 1.17 [95% CI, 1.05–1.31], *P* < 0.001) were independent factors associated with PCL rupture.

**Conclusion:**

Through this retrospective observational study, we found that patients with PCL rupture may have a higher posterior intercondylar eminence compared to PCL-intact patients.

**Level of evidence:**

III.

## Background

The posterior cruciate ligament (PCL) acts to restraint translation of the posterior tibia relative to the femur at the knee [1]. The incidence of PCL injury has increased in the past decades, possibly due to more people participating in sports and playing more intensively, as well as increased traffic accidents [2]. Although it is less common than anterior cruciate ligament (ACL) injury, the PCL is susceptible to injury from a posterior force directed onto the proximal tibia, most commonly with the knee in a flexed position [3–5]. This often occurs in patients who fall onto a flexed knee with the foot plantar flexed, such that the proximal tibia strikes the ground first [6]. Although the biomechanical mechanisms of PCL tears have been well described, there is still little research regarding the anatomic geometry of the knee joint and its association with PCL injury risk [7].

**Fig. 1:**
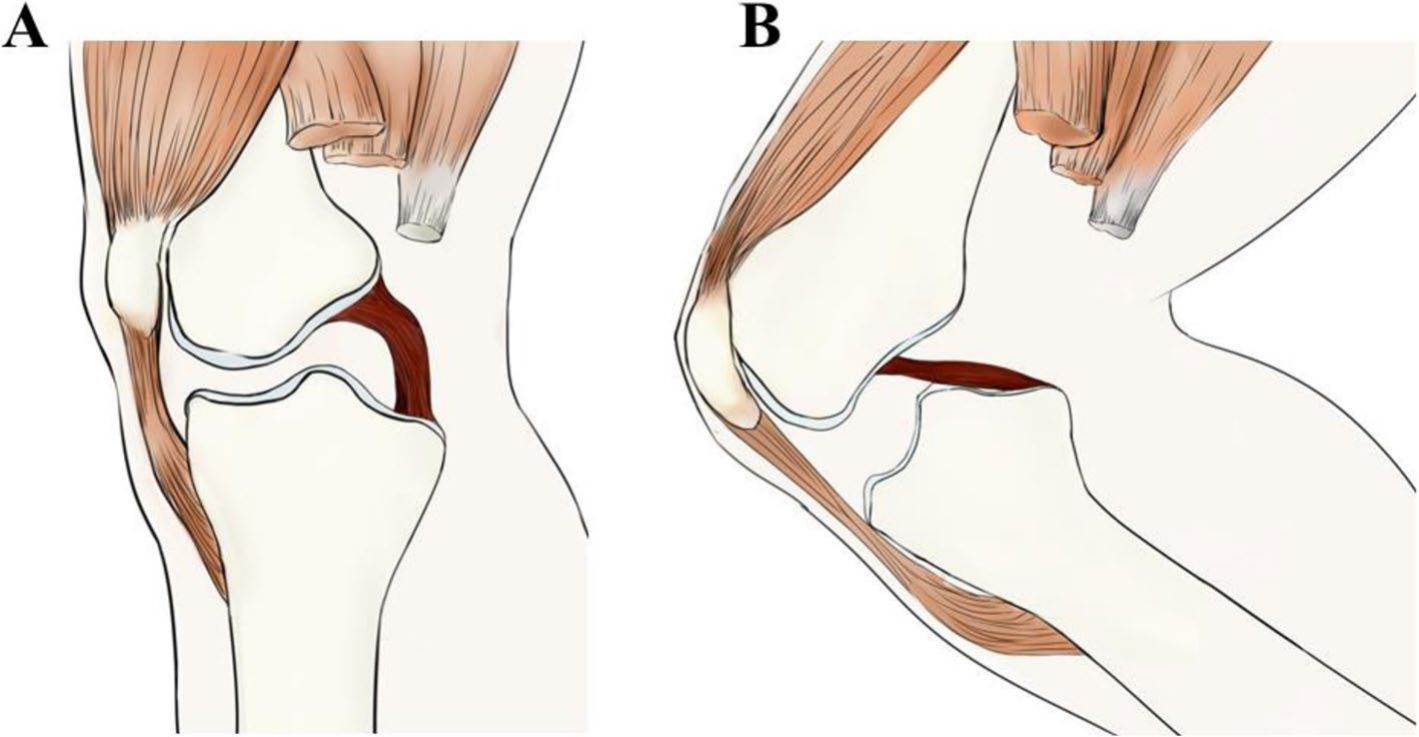
Schematic diagram of normal PCL position in (**A**) knee extension and (**B**) knee flexion

A recent study found that patients with PCL rupture had higher tibial eminences than PCL-intact patients on anterior–posterior X-ray [8]. In knee extension, the PCL is concave antero-inferiorly and is relatively distant from the posterior intercondylar eminence (PIE). As the knee flexes, the PCL gradually straightens and moves closer to the PIE [9, 10]. It has been reported that the maximal length of the PCL in the living knee is at 90–100° of knee flexion and the PCL is closest to the apex of the PIE at this time (Fig. 1) [9–11]. If the PIE is too high, it may cause the tight PCL to collide with the bone and rupture if a sudden posteriorly directed force is applied to the proximal tibia at knee flexion when the PCL bears maximum tension [1, 10]. However, the previous study just used a statistical shape model in X-ray and did not specifically measure the height of tibial PIE. In this study, we will evaluate the relationship between the height of tibial PIE and PCL rupture by measuring on MRI and hypothesized that higher PIE may be a risk factor for PCL rupture due to the impingement mechanism during the flexion of the knee.

## Methods

This case–control study on the risk factors of PCL rupture was carried out in our sports medicine institute. And it was approved by the Ethics Committee of the Third Hospital of Peking University. All methods were performed in accordance with the guidelines and regulations of the Ethics Committee of the Third Hospital of Peking University.

Patients who underwent PCL reconstruction from 2015 to 2018 were retrospectively analyzed. All the patients with PCL rupture received MRI examination and operation within 6 months after injury. The patient’s history, results of physical examination and MRI were used to confirm the diagnosis and determine any multiple injury. The inclusion criteria were:(1) 16 ≤ age ≤ 55, (2) MRI images in our PACS system, (3) isolated PCL injury, (4) rupture of PCL defined by MRI, (5) Sports enthusiasts and the injury occurred in knee flexion during exercise. Exclusion criteria were: (1) avulsion fracture of the intercondylar eminence, (2) PCL avulsion fractures, (3) history of tibial fractures, (4) prior osteotomy, (5) concomitant PCL and ACL rupture, (6) detectable osteoarthritis, (7) traffic accidents.

The control group was PCL-intact inpatients diagnosed with ACL rupture who underwent ACL reconstruction because of a sports-related accident during the same period. This group showed no evidence of ligament backward instability on physical examination by a professional sports medicine doctor, and no evidence of PCL rupture on MRI. Patients were excluded if they had any congenital or pathologic condition known to affect PIE-related data measurement, including but not limited to history of tibial fractures, tibial osteotomy procedures, rheumatoid arthritis and congenital genu recurvatum, which may change their native bony geometry.

According to our inclusion criteria and exclusion, we included 55 patients with unilateral PCL rupture as case group and no patient had bilateral PCL rupture. We chose 328 patients with ACL rupture as control group. To control the baseline conditions between two groups and minimize the effect of potential confounding factors, a 1-to-1 propensity score matching was performed. Variables included for the matching were age, gender, height, weight, and side of injury. A caliper of 0.06 was utilized in the match, and replacement of patients in the algorithm was not allowed. After propensity score matching, all variables were successfully matched, without statistically significant differences. Finally, in each group, 55 patients were enrolled for the analysis.

### Imaging evaluation

Clinical examination, MRI and arthroscopic procedures were reviewed to confirm the presence of PCL rupture by two sports medicine surgeons. The PCL-intact control group was also reviewed to confirm patient information, especially the lack of PCL rupture or detectable osteoarthritis in MRI images by two observers. All of the basic information (name, age, sex, diagnosis and MRI reports) was removed. Two independent observers (fellowship of sports medicine) were trained in measuring the data in the PACS system and were blinded to each other's measurements and the hypothesis for the study. Neither of the observers was involved in the treatment of the patients or had any knowledge of their clinical data. The results measured by two observers were reported using intraclass correlation coefficients(ICCs) with their corresponding 95% confidence intervals (CIs) [12]. The ICC values were interpreted as follows: ICC of > 0.75: excellent agreement; ICC of 0.40–0.75: fair-to-good agreement; and ICC of < 0.40: poor agreement [13]. The average of the two measurements were used for each group to decrease potential measurement bias.

**Fig. 2:**
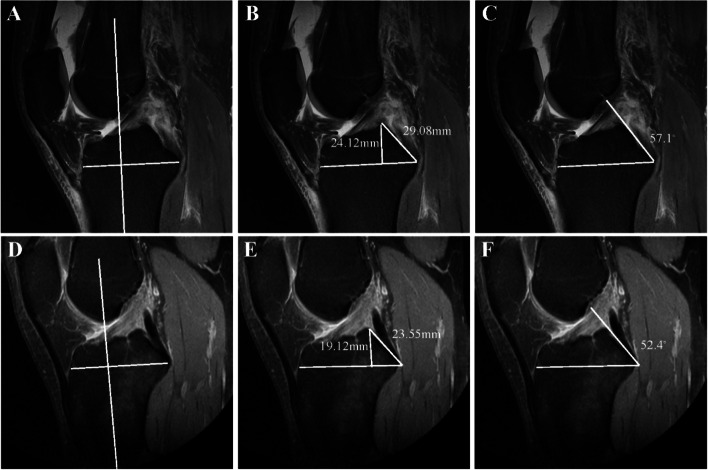
**A**-**C**: Measurements of HPIE, SLPIE and SAPIE in PCL rupture patient. **D**-**F**: Measurements of HPIE, SLPIE and SAPIE in PCL-intact patient. A and D, reference line. **B** and **E**, HPIE was measured as the perpendicular length from the apex to the reference line, and SLPIE was measured as the distance from the apex to the lowest tibial attachment of the PCL **C** and **F**, SAPIE was measured between the reference line and the tangent of the slope of the posterior intercondylar eminence

The height of the apex of the posterior intercondylar eminence (HPIE), the slope length (SLPIE) and the slope angle of the posterior intercondylar eminence (SAPIE) were measured using conventional MRI scans from a T2 sequence by using a 1.5-T MRI scanner. The slice thickness of MRI was 4 mm. The first step was to define the sagittal-plane longitudinal central axis of the tibia, which consisted of the tibial attachment of the PCL and ACL and the intercondylar eminence. This central axis was confirmed by determining the midpoint of the anterior to posterior borders of the tibia at two points located as distally as possible on the tibial tuberosity in the MRI image. The line connecting the two midpoints represents the longitudinal central axis of the tibia in the sagittal plane [14]. A perpendicular reference line was drawn from the lowest tibial attachment of the PCL to the longitudinal central axis in the sagittal plane. The HPIE was then measured as the perpendicular length from the apex to the reference line. The SLPIE was then measured as the distance from the apex of posterior intercondylar eminence to the lowest tibial attachment of the PCL. The SAPIE was measured as the angle between the reference line and the tangent of the slope of the posterior intercondylar eminence, which was defined as the line connecting the two uppermost sites on the slope (Fig. 2).

### Statistical analysis

Independent sample t tests were performed to compare the differences in patient age, height and BMI between the PCL rupture case group and the PCL-intact control group.

A Chi-square test was used to compare differences of gender and limb side between groups. Measurements were expressed in mean ± standard deviations (SD). Independent sample t tests were used to compare mean HPIE, SLPIE and SAPIE between groups. Logistic regression analyses were used to evaluate confounding variables, and odds ratios (ORs) was used to identify risk factors. All statistical analysis was performed using SPSS Statistics 27(IBM), with *P* < 0.05 considered statistically significant.

## Results

Patient characteristics are presented in Table 1. In summary, the case group consisted of 43 men and 12 women, and the control group consisted of 43 men and 12 women. There was no significant difference in gender (*P* = 1.000), limb side (*P* = 0.848) between the case and control group by Chi-square testing. There was also no significant difference in age (*P* = 0.291), BMI (*P* = 0.444) or height (*P* = 0.290) between the groups by Independent samples t tests (Table 1).

**Table 1:** Clinical characteristics of patients with PCL rupture and intact controls

	Case group	Control group	*P* value
Patients(knees)	55 (55)	55 (55)	
Gender(male/female)	43/12	43/12	1.000
Limb side(left/right)	26/29	25/30	0.848
Height (cm), mean ± SD	174.60 ± 8.12	172.98 ± 7.84	0.290
BMI (kg/m^2^)	24.13 ± 2.87	24.58 ± 3.33	0.444
Age (y) mean ± SD	29.76 ± 12.53	31.98 ± 9.09	0.291

The reproducibility of the measurements is shown in Table 2. Inter-observer reproducibility was excellent agreement in HPIE, SLPIE and SAPIE of case and control groups (ICC: HPIE = 0.81, SLPIE = 0.77, SAPIE = 0.85).

**Table 2:** Inter-observer reproducibility of the measurements by two observers

	ICC	ICC 95% CI
HPIE	0.81	0.73–0.86
SLPIE	0.77	0.68–0.83
SAPIE	0.85	0.79–0.90

The case group of 55 PCL-ruptured patients had a mean HPIE of 19.76 ± 2.29 mm, with a mean HPIE of 20.21 ± 2.20 mm in male group (*n* = 43) and 18.16 ± 1.89 mm in female group (*n* = 12). The control group of 55 PCL-intact patients had a mean HPIE of 17.32 ± 1.80 mm, with a mean HPIE of 17.50 ± 1.66 mm in male group (*n* = 43) and 16.69 ± 2.19 in female group (*n* = 12). The study group had a mean SLPIE of 24.30 ± 2.38 mm, with a mean SLPIE of 24.95 ± 2.22 mm in male group (*n* = 43) and 21.97 ± 1.18 mm in female group (*n* = 12). The control group had a mean SLPIE of 22.98 ± 2.10 mm, with a mean SLPIE of 23.05 ± 2.01 mm in male group (*n* = 43) and 22.75 ± 2.45 in female group (*n* = 12). The study group had a mean SAPIE of 53.06° ± 4.55°, with a mean SAPIE of 52.81° ± 4.81° in male group (*n* = 43) and 53.93° ± 3.47° in female group (*n* = 12). The control group had a mean SAPIE of 48.54° ± 4.23°, with a mean SAPIE of 49.00° ± 4.20° in male group (*n* = 43) and 46.92° ± 4.11° in female group (*n* = 12) (Table 3).

**Table 3:** Anatomic geometry of the posterior intercondylar eminence

	Case group	Control group	*P* value
HPIE (mm)	19.76 ± 2.29	17.32 ± 1.80	< 0.001
Male	20.21 ± 2.20	17.50 ± 1.66	< 0.001
Female	18.16 ± 1.89	16.69 ± 2.19	0.092
SLPIE (mm)	24.30 ± 2.38	22.98 ± 2.10	0.003
Male	24.95 ± 2.22	23.05 ± 2.01	< 0.001
Female	21.97 ± 1.18	22.75 ± 2.45	0.332
SAPIE (°)	53.06 ± 4.55	48.54 ± 4.23	< 0.001
Male	52.81° ± 4.81°	49.00° ± 4.20°	< 0.001
Female	53.93° ± 3.47°	46.92° ± 4.11°	< 0.001

Patients with PCL rupture had significantly greater mean HPIE, SAPIE (both *P* < 0.001), and SLPIE (*P* < 0.05) than control patients. Multivariable analysis showed that HPIE (OR, 1.62 [95% CI, 1.24–2.11], *P* < 0.001) and SAPIE (OR, 1.17 [95% CI, 1.05–1.31], *P* < 0.001) were independent factors associated with PCL rupture (Table 4).

**Table 4:** Multivariable Logistic Models for risk factors associated with PCL rupture

	OR (95%CI)	*P* value
HPIE (mm)	1.62(1.24–2.11)	< 0.001
SAPIE	1.17 (1.05–1.31)	0.006

## Discussion

In this study, higher HPIE and greater SAPIE in the PCL-ruptured group were found compared to the PCL-intact group and it may be risk factors for the PCL rupture in knee flexion injury. In addition, patients with PCL injury had significantly greater SLPIE than PCL-intact patients.

The PCL originates from the anterolateral aspect of the medial femoral condyle and inserts onto the posterior aspect of the tibial plateau. It is the most important ligament in maintaining the posterior stability of the knee joint [15, 16]. It can be separated into the anterolateral and the posteromedial bundles [17, 18], of which the anterolateral bundle (65% of the body of the PCL) is about twice as large as the posteromedial bundle in cross-sectional area [15, 18, 19]. The anterolateral bundle is taut during knee flexion and lax in extension; conversely, the posteromedial bundle is taut during extension and lax in flexion [15, 18]. MRI of the knee in flexion shows the PCL gradually approaching the posterior intercondylar eminence [9, 10]. Therefore, we chose PCL-ruptured cases when its mechanism is knee flexion for this study. According to the biomechanical mechanism of the PCL, the anterolateral portion is commonly injured in knee flexion. In addition, the spectrum of PCL injuries includes intra-substance injury, complete substance tears and avulsion of the PCL insertion sites on the femur and tibia [11, 19]. In our study, substance tears were incorporated because of the injury mechanism.

Previous studies have focused on the bony morphology of the knee as a risk factor in ACL-ruptured patients [14, 20–22]. However, to our knowledge, few studies have focused on the anatomical risk factors for PCL rupture [7, 8]. A recent study found that patients with PCL rupture had higher tibial eminences than PCL-intact patients on anterior–posterior X-ray [8]. However, that study just used a statistical shape model in X-ray and did not specifically measure the height of the tibial intercondylar eminence. In addition, it only described this phenomenon of higher tibial eminences resulting in PCL injuries without exploring the possible mechanism. In our study, we defined the HPIE, SLPIE and SAPIE and measured the specific data on MRI in PACS system (Fig. 2). The measurement method refers to other previously-published studies on the relationship between tibial slopes and ACL injuries on MRI [14].

In extension of the knee, the PCL is curved concave-forwards away from the posterior intercondylar eminence. It is straight, fully out-to length with the knee at 90° flexion and close to the posterior intercondylar eminence (Fig. 1). In full flexion, it curves convex-forwards over the roof of the intercondylar notch [9, 10]. It has been reported that in situ force in the PCL increases with knee flexion ranging from 35.6 ± 13 N at 0° flexion to 112.3 ± 28.5 N at 90° flexion, in response to 95% of posterior translational forces at 90° [6, 10, 23]. In addition, studies have found that the maximal length of the PCL in the living knee is at 90–100° of knee flexion and the PCL is closest to the posterior intercondylar eminence apex at this time [9–11]. At this time, a sudden posteriorly-directed force applied to the proximal tibia may cause collisions between the bony structures and stretched ligaments. In this study, we found that the mean HPIE, SLPIE and SAPIE of patients with PCL rupture were significantly greater than PCL-intact patients. HPIE and SAPIE were independently associated with PCL rupture using multivariable logistic analyses regression model which means that PCL is closer to the posterior intercondylar eminence apex in PCL-ruptured patients. In this study, as well as matching age, gender, height, weight, and side of injury, we specifically chose patients with knee trauma as the control group to make the groups more comparable than using healthy controls without a traumatic event. Research into anatomical risk factor for PCL rupture is rare. In a previous study, Kuijk [8] found that a more sharply angled intercondylar notch is related to a PCL rupture. In this study, we found that patients with PCL rupture may have a higher posterior intercondylar eminence compared to PCL-intact patients. It may be helpful for protecting the graft when polishing the high HPIE during posterior cruciate ligament reconstruction. Also, we think the anatomical risk factor may help to identify individual patients who are at great risk of PCL rupture.

### Limitations

We acknowledge some limitations to our study. In this study, the PCL injured group were only those that had PCL reconstruction performed so that the results may not be generalizable to the non-operatively managed PCL injured patients. Another limitation is that the theory of collision between the PCL and posterior intercondylar eminence during knee flexion was inferred from clinical data. There are no biomechanical studies on cadavers to directly explain the phenomenon. Third, we have chosen patients with ACL injuries but PCL-intact as the control group in this study, which may cause some misleading that lower HPIE, SLPIE and SAPIE were risk factors for ACL injuries. In terms of anatomical positional relationship, the posterior intercondylar eminence and ACL will not collide theoretically. However, it still needs biomechanical studies of cadavers to confirm this. In addition, patients in control group may not experience a similar mechanism of injury. And, we selected patients with sports injuries during the same period as controls to minimize errors. Fourth, there were no radiographs of uninjured knees in our study. The uninjured knees of patients in our study have no symptoms and they refused MRIs of uninjured knees. Thus, the parameters could not be measured to examine whether there are significant differences between the involved knees and uninjured knees.

## Conclusion

Through this retrospective observational study, we found that patients with PCL rupture may have a higher posterior intercondylar eminence compared to PCL-intact patients.

## Data Availability

The datasets analysed during the current study is available from the corresponding author on reasonable request.

## Abbreviations

PCL: Posterior cruciate ligament
ACL: Anterior cruciate ligament
PIE: Posterior intercondylar eminence
ICCs: Intraclass correlation coefficients
HPIE: Posterior intercondylar eminence
SLPIE: Slope length of the posterior intercondylar eminence
SAPIE: Slope angle of the posterior intercondylar eminence
